# Machine learning in predicting outcomes for stroke patients following rehabilitation treatment: A systematic review

**DOI:** 10.1371/journal.pone.0287308

**Published:** 2023-06-28

**Authors:** Wanting Zu, Xuemiao Huang, Tianxin Xu, Lin Du, Yiming Wang, Lisheng Wang, Wenbo Nie

**Affiliations:** School of Nursing, Jilin University, Changchun, China; Houston Methodist Academic Institute, UNITED STATES

## Abstract

**Objective:**

This review aimed to summarize the use of machine learning for predicting the potential benefits of stroke rehabilitation treatments, to evaluate the risk of bias of predictive models, and to provide recommendations for future models.

**Materials and methods:**

This systematic review was conducted in accordance with the PRISMA statement and the CHARMS checklist. The PubMed, Embase, Cochrane Library, Scopus, and CNKI databases were searched up to April 08, 2023. The PROBAST tool was used to assess the risk of bias of the included models.

**Results:**

Ten studies within 32 models met our inclusion criteria. The optimal AUC value of the included models ranged from 0.63 to 0.91, and the optimal R^2^ value ranged from 0.64 to 0.91. All of the included models were rated as having a high or unclear risk of bias, and most of them were downgraded due to inappropriate data sources or analysis processes.

**Discussion and conclusion:**

There remains much room for improvement in future modeling studies, such as high-quality data sources and model analysis. Reliable predictive models should be developed to improve the efficacy of rehabilitation treatment by clinicians.

## Introduction

Stroke remains one of the most common diseases that causes functional impairment, especially due to the rapidly growing number of older adults [1]. Due to the increasing prevalence of patients suffering from the effects of stroke, the importance and burden of stroke rehabilitation are high [1, 2]. In recent years, many effective stroke rehabilitation treatments have been proposed through randomized trials, such as task-oriented training treatment, functional strength training, and robot-assisted treatment [3–5]. Nonetheless, clinicians often face the challenge of choosing the most adequate rehabilitation treatment for patients since the benefits of treatments vary across individuals with different characteristics [6]. The precise prediction of rehabilitation treatment is therefore important for properly distributing rehabilitation resources and delivering patient-specific rehabilitation [7, 8].

Machine learning is a type of artificial intelligence that focuses on constructing computerized algorithms to automatically improve performance through experience. In recent decades, machine learning has shown an ability to effectively deal with high-throughput data, and it has become a popular method in many fields, ranging from biology to social science [9, 10]. Many kinds of research based on machine learning have also evolved in the medical field due to its ability to handle health care data and thus aid clinical workflows. In the stroke field, machine learning methods are currently applied in early detection, diagnosis, and outcome prediction [11, 12]. Recently, an increasing number of studies have examined machine learning methods with the aim of predicting outcomes and identifying stroke patients who might benefit from specific rehabilitation treatments. A systematic review that evaluates the quality of these studies would be beneficial for further similar studies.

### Objective

This review aimed to systematically summarize studies that used machine learning methods to build models as well as externally validated studies that predicted the potential benefits of patients following stroke rehabilitation treatments. We also aimed to evaluate the risk of bias of the included models and therefore propose potential improvements, which might provide evidence for further modeling studies and thus aid the decision-making process in stroke rehabilitation clinical settings.

## Materials and methods

### Protocol

This review was performed in accordance with the PRISMA statement and the **CH**ecklist for critical **A**ppraisal and data extraction for systematic **R**eviews of prediction **M**odelling **S**tudies (CHARMS) [13, 14]. The CHARMS checklist was developed to support the design of systematic reviews of predictive modeling studies and provides guidance for forming the review question, study selection, and data extraction. The aim of our review was summarized into key items, as presented in Table 1. In addition, our systematic review has been registered on PROSPERO (ID number: CRD42022299195, available at https://www.crd.york.ac.uk/PROSPERO/).

**Table 1:** CHARMS guidelines for the formation of review question.

Key item	Definition
Intended scope of the review	Studies aimed at predicting clinical outcomes after specific rehabilitation treatment for stroke patients
Type of prediction modelling studies	Model development or validation studies
Target population to whom the prediction model applies	Post-stroke patients that had received rehabilitation treatment
Outcome to be predicted	Motor functional outcomes measured by standardized scales
Intended moment of using the model	Before rehabilitation treatment

Table 1 shows the aim of this review according to the CHARMS guidelines.

### Inclusion and exclusion criteria

Given the aim of this review, the eligibility criteria were as follows:

Inclusion criteria

· Studies focused on the development or validation of prediction models for recovery potential after stroke rehabilitation

· Models based on machine learning methods

· Patients in the primary studies must have received specific stroke rehabilitation treatment regardless of the stroke stage and age of the patients

· The predicted outcomes of the model must be motor functional outcomes assessed through standard tools

· The prediction model was designed for use before rehabilitation treatment

Exclusion criteria

· Studies aimed at identifying predictors related to outcomes rather than predicting clinical outcomes for individual patients

· Studies aimed at evaluating the impact of using predictive models in clinical settings

· Full-text article was not available

· Model methods were not reported in detail, including study protocol, conference abstracts, letters, etc.

· Reviews or comments without original research

### Search strategy

Two authors independently searched the PubMed, Embase Cochrane Library, Scopus, and CNKI (China National Knowledge Infrastructure) databases up to December 15, 2021 (updated on April 08, 2023) to identify relevant studies. ‘Stroke’, ‘machine learning’, ‘rehabilitation’, and their synonyms were used as MeSH terms or free-text words to identify eligible studies. An example search strategy for PubMed is provided in the S1 Table. We also manually searched the reference lists and citations of the included studies as well as Google Scholar to obtain additional resources.

After removing duplicates, we selected eligible studies based on titles and abstracts in accordance with the inclusion and exclusion criteria described above. The full texts were then screened by two reviewers, and any disagreements were resolved by consulting a third reviewer.

### Data extraction and quality assessment

A data extraction sheet was used to address any information that would increase the risk of bias of the models. Briefly, the extracted data included the source of data, participants, predicted outcomes, predictors, model development, model performance, and model evaluation methods, as recommended in the CHARMS checklist [14]. We extracted discrimination and calibration data as primary metrics to describe model performance. Discrimination is often estimated by the area under the receiver-operating characteristic curve (AUC-ROC) for logistic regression models and should reflect the ability of a model to distinguish between individuals with or without the outcome of prediction models. Calibration is often estimated by calculating the Hosmer–Lemeshow goodness-of-fit test with a calibration pot and should reflect the agreement between the predicted and the observed outcome [15, 16]. We entered the details into the data extraction sheet, which is provided in the S2 Table.

PROBAST (Prediction model Risk Of Bias ASsessment Tool) was used to guide the risk of bias assessment in this review [15]. The PROBAST tool was mainly designed to estimate the quality of the individual prediction model in systematic reviews. The prediction models were explicitly classified into three types in this tool and relevant signaling questions were proposed for evaluating different types of prediction models. Furthermore, the signaling questions were grouped into four domains of the potential source of bias: participants, predictors, outcome, and analysis. If one of the four domains had high risk of bias (ROB), the overall judgement would be a high ROB [17]. The unit of evaluation applied in this review was model rather than study, since some studies might develop or validate several models.

## Results

### Search results

**Fig 1:**
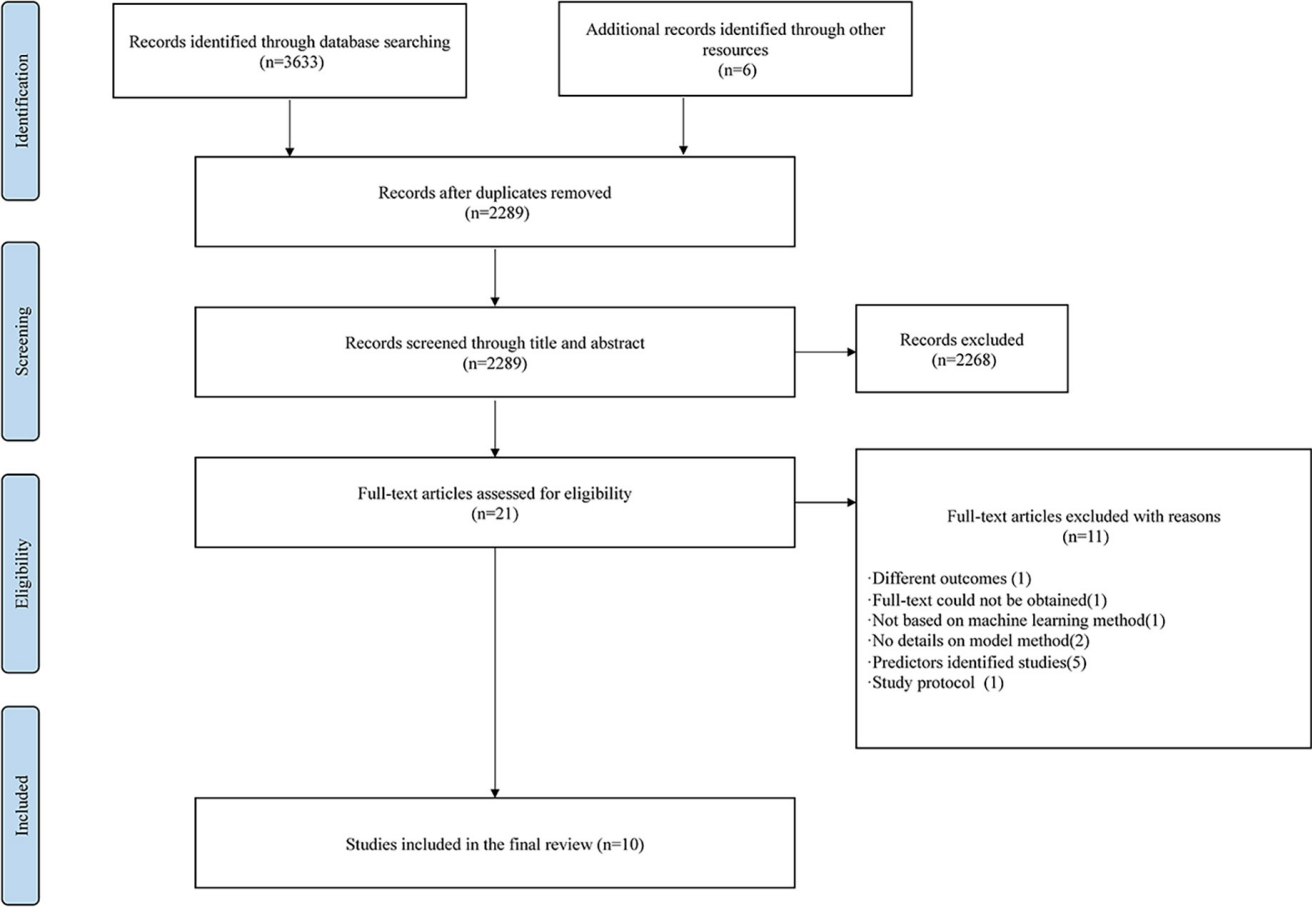
PRISMA flow chart. Fig 1 shows the selection process of eligible studies in this review according to PRISMA.

The PRISMA flowchart (Fig 1) presents the selection process of eligible studies in this review. In total, 3639 records were obtained based on the search strategy. After deleting duplicates, 2289 records were then screened according to the title and abstract. The majority of studies were excluded at this stage because their aims, designs, and outcomes were outside the scope of this review. Twenty-one full-text studies that met the inclusion and exclusion criteria were then examined and excluded for the reasons shown in the flowchart. Finally, 10 studies were included in this narrative review, and 32 models were included in the risk of bias assessment.

### Characteristics of included studies

We summarized the characteristics of the included studies in Table 2. All ten studies described model development, and one of them also considered external validation for previous models [18]. Only two studies used data from randomized trials [19, 20], whereas the majority of the studies used electronic medical records as the data source for model development or validation. Five studies utilized multicenter data within their country as data sources [19–23]. Four of the included articles were conducted in the United States [19, 22–24], four were conducted in Europe [18, 21, 25, 26], and two were conducted in Asia [20, 27].

**Table 2:** Characteristics of included studies.

Study	Number of models	Study type	Data source	Sample size Stroke stage	Rehabilitation treatment	Outcome	Number and type of predictors in the final model	Model development method	Optimal model performance results
Tozlu et al., 2020	10	Model development	Multicenter randomized trial	N = 102 Chronic stroke	Repetitive TMS and physical therapy	Post-intervention UE-FMA	93 Demographic variables, clinical variables, 2 TMS-based neurophysiological measures, and structural disconnectivity measurements for 86 regions	EN ANN SVM CART RF	R^2^ 0.910 RMSE 0.362
						FMA changed <5.5 (nonresponder) or ≥5.5 (responder) MCID = 5.5	93 Demographic variables, clinical variables, 2 TMS-based neurophysiological measures, and structural disconnectivity measurements for 86 regions	EN ANN SVM CART RF LR	AUC 0.63
Scrutinio et al., 2017	2	Model development	Electronic medical records	Derivation Cohort (n = 717); Validation Cohort (n = 875) Acute and subacute stroke	Physical and occupational therapy	M-FIM score of >61 points at discharge	5 Age, Time since onset, Unilateral neglect, Motor-FIM score, Cognitive FIM score	LR	AUC 0.883 HL test 4.12 (P = 0.249)
						physical independence grade ≥5 (FIS)	5 Age, Time since onset, Male gender, Motor-FIM score, Cognitive FIM score	LR	AUC 0.913 HL test 1.20 (P = 0.754)
Lee et al., 2021	2	Model development	Electronic medical records	N = 86 Subacute stroke	Robot-assisted therapy using InMotion2 along with conventional therapy	FMA changed≥9 (responder) MCID = 9	2 Time since onset Hand Movement Scale	LR	AUC 0.658
				N = 58 Chronic stroke		FMA changed≥5.25 (responder) MCID = 5.25	2 Time since onset Hand Movement Scale	LR	AUC 0.739
García-Rudolph et al., 2021	2	External validation	Electronic medical records	N = 710 Acute and subacute stroke	Inpatient rehabilitation program	M-FIM score of >61 points at discharge	5 Age, Time since onset, Unilateral neglect, Motor-FIM score, Cognitive FIM score	LR	AUC 0.873 HL test 6.07 (P = 0.63)
		External validation	Electronic medical records	N = 710 Acute and subacute stroke	Inpatient rehabilitation program	physical independence grade ≥5 (FIS)	5 Age, Time since onset, Male gender, Motor-FIM score, Cognitive FIM score	LR	AUC 0.803 HL test 8.91 (P = 0.34)
		Model development	Electronic medical records	N = 710 Acute and subacute stroke	Inpatient rehabilitation program	M-FIM score of >61 points at discharge; physical independence grade ≥5 (FIS)	5 Age, Time since onset, Unilateral neglect, Motor-FIM score, Aphasia	LR	AUC 0.894 HL test 10.40 (P = 0.23) for primary outcome AUC 0.845 HL test 6.94 (P = 0.54) for secondary outcome
Bates et al.,2015 PART 1	1	Model development	Electronic medical records	N = 4020 No limitation	consultative or comprehensive treatment	physical grade IV (FIM)	7 Age, clinical variables, type of rehabilitation, recovery time, acute procedure	LR	AUC 0.84 HL test P = .93
Bates et al.,2015 PART 2	1	Model development	Electronic medical records	N = 5416 No limitation	consultative or comprehensive treatment	physical grade VI (FIM)	7 Age, clinical variables, type of rehabilitation recovery time, acute procedure	LR	AUC 0.83 HL test P = .38
Harari et al, 2020	4	Model development	Electronic medical records	N = 50 No limitation	Inpatient rehabilitation program	FIM	10 Demographic information, stroke characteristics, and scores of the clinical tests	Lasso regression	R^2^ 0.76 MAE 7.6
						Ten-Meter Walk Test	6 Demographic information, stroke characteristics, and scores of the clinical tests		R^2^ 0.70 MAE 0.26
						Six-Minute Walk Test	4 Demographic information and scores of the clinical tests		R^2^ 0.70 MAE 73.2
						Berg Balance Scale	8 Demographic information, stroke characteristics, and scores of the clinical tests		R^2^ 0.77 MAE 6.4
Thakkar et al., 2020	2	Model development	Randomized controlled trials	N = 239 Chronic stroke	Contemporary task‑oriented interventions	FMA changed≥4 (high responders) FMA changed<4 (low responders) MCID = 4	3 Time since stroke, baseline FIM scores and FMA scores	KNN ANN	Accuracy 85.42% AUC 0.89
Goffredo et al., 2022	6	Model development	Electronic medical records	N = 66 Subacute stroke	InMotion2 based upper limb Robot-assisted Therapy (ulRT) along with conventional therapy	Post-intervention MI_ELBOW_	4 RMK metrics	MLR	R^2^ 0.683 RMSE 5.547
						Post-intervention MI_SHOULDER_	4 RMK metrics		R^2^ 0.640 RMSE 5.294
						Post-intervention MI_UL_	4 RMK metrics		R^2^ 0.765 RMSE 13.859
Gandolfi et al, 2023	2	Model development	Electronic medical records	N = 95 Subacute stroke	tailored upper limb rehabilitation treatment	UE-FMA at discharge	24 demographic and clinical domains; cognitive; body function and disability domains	MLR RF	R^2^ 0.807 RMSE 6.17

TMS, transcranial magnetic stimulation; MCID, minimal clinically important difference; UE-FMA, upper-extremity Fugl-Meyer Assessment; FIM, Functional Independence Measure; FIS, Functional Independence Staging system; ANN, artificial neural network; CART, classification and regression trees; EN, elastic net; RF, random forest; SVM, support vector machine; KNN k-nearest neighbors; LR, logistic regression; RMSE, root of mean squared error; AUC, area under the receiver operating characteristic curve; HL test, the Hosmer–Lemeshow test; MAE, Mean Absolute Error; MI_ELBOW_, Motricity Index affected elbow flexion; MI_SHOULDER_, Motricity Index affected shoulder abduction; MI_UL_, Motricity Index affected Upper Limb; RMK, Robot-Measured Kinematic; MLR, multiple linear regression; SSV, Split Sample Validation; LOOCV, Leave-One-Out Cross-Validation.

Table 2 described the characteristics of included studies.

Regarding the participants included in the primary studies, two studies selected chronic stroke as one of the inclusion criteria for patients [19, 20], and two studies only included patients who had been admitted within 90 days of onset of stroke [18, 21]. Two studies included subacute phase stroke patients [25, 26], whereas the remaining four studies had no restrictions on the stage or type of stroke; however, one study explicitly excluded acute stroke patients [27]. Furthermore, all of the participants had completed an organized physical rehabilitation program, one of the studies involved transcranial magnetic stimulation [19], and two studies used robot-assisted rehabilitation [25, 27].

Among the included studies, regression was the most common method implemented to develop models. Specifically, logistic regression was used in six studies [18, 19, 21–23, 27], linear regression was used in two studies [25, 26], and a single study used Lasso regression [24]. Other common machine learning approaches, such as artificial neural network, k-nearest neighbors, and random forest were used in three studies [19, 20, 26], as presented in Table 2. Four studies provided models with external validation [20–23], four studies considered internal validation [19, 24–26], and a single study did not mention the validation process [27]. Moreover, one study externally validated two existing models in a previous study using data from a different country and also developed a novel model with internal validation using the same database [18]. The optimal AUC value of the included models ranged from 0.63 to 0.91. Four studies chose the R^2^ value to describe the discrimination of models, and it ranged from 0.64 to 0.91 [19, 24–26]. These outcomes suggest that the discriminative ability of the included models varied.

### Quality assessment of included studies

**Fig 2:**
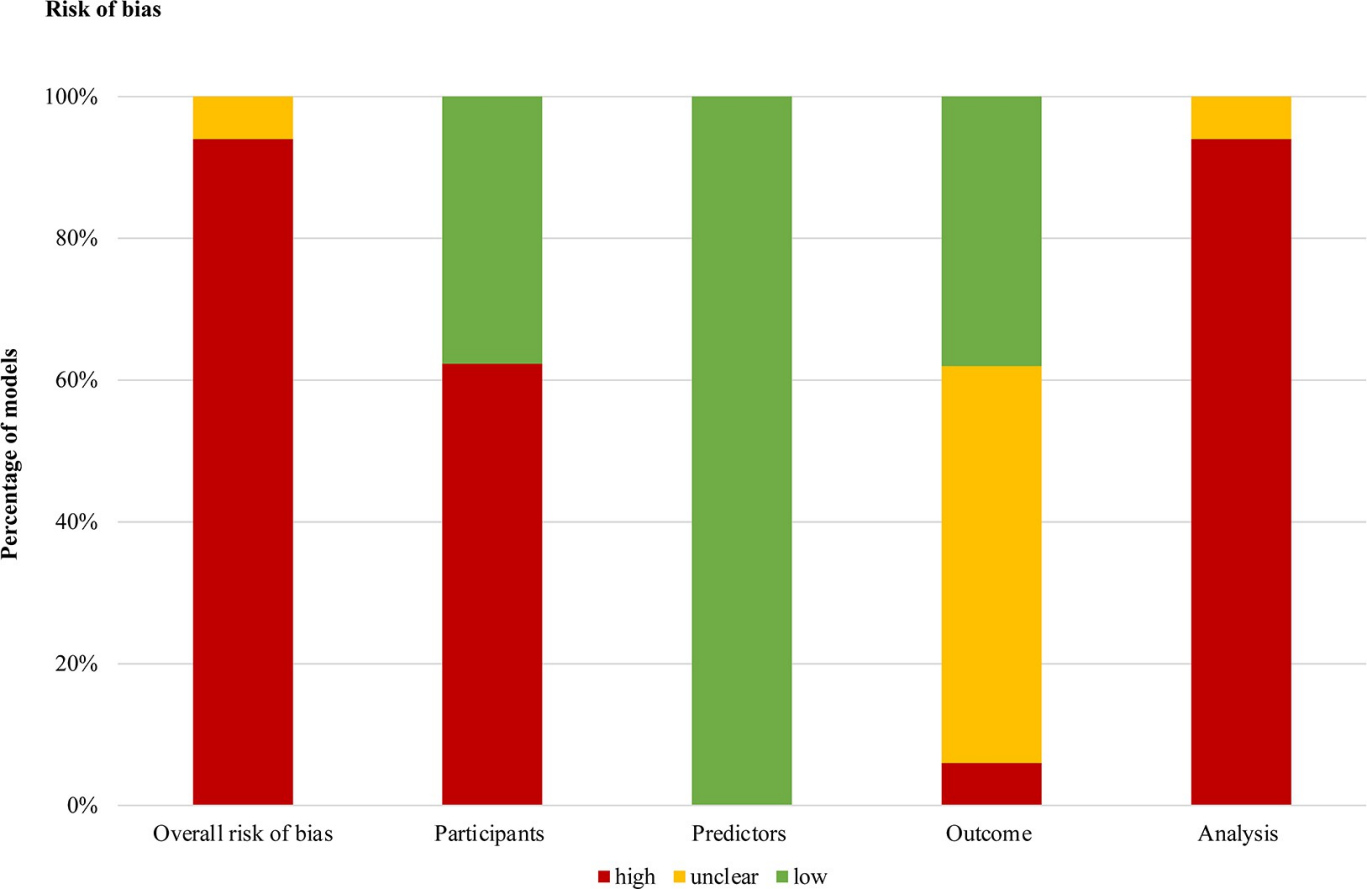
Risk of bias summary. Fig 2 shows the percentage risk of bias ratings for each aspect of the included models according to the PROBAST tool." high", "unclear" and "low" represent a high, unclear, and low risk of bias, respectively.

According to the PROBAST tool, all the models demonstrated an overall high (n = 30) or unclear (n = 2) risk of bias (Fig 2). This indicates that the performance and usability of each model might be overoptimistic. Nearly all the models were biased from participants and analysis domains, and the common causes of downgrading were inappropriate data sources or analysis processes. Models from the same study have a common risk of bias in terms of participant domain since they share the same data source. Among the included models, only twelve models in two studies that used randomized trail data were rated as having a low risk of bias in the participant domain. Twenty models were rated as an unclear risk of bias or high risk of bias in the outcome domain. All the models had a low risk of bias for the predictor domain, which indicates that all the predictors selected could be obtained before treatment and tend to be assessed in similar ways. However, in the analysis domain, all the remaining models were rated as having a high risk of bias, except two models were considered to have an unclear risk of bias.

## Discussion

In recent years, as machine learning has emerged as an attractive approach to address big data in health care, many related studies have bene published, especially studies of stroke patients. In this review, we systematically searched studies aimed at using machine learning methods to predict recovery potential following stroke rehabilitation treatments. Based on our results, we will discuss the possible bias of the included models and its impact from the four important aspects of constructing models specified by the PROBAST tool, and we will suggest future research directions.

### Participants

Most of the included studies used electronic medical records (EMRs) as the data source for prediction model development; however, the inherent biases of EMRs should be noted [17]. For example, since routine care data are usually measured by general practitioners, measurement definitions may differ between individuals, particularly among multicenter practitioners [28, 29]. While data from randomized control trials are usually the gold standard for collecting data, they may not always reflect the real world due to their narrow inclusion criteria [30]. Thus, leveraging both interventional data from trials and observational data from the real world could be considered for further studies [31, 32]. Furthermore, stroke type and stage were not restricted and classified in some studies we reviewed. Although a larger scope of the target population would increase the generalizability of models, confounders could also increase to some extent. For example, an ambiguous time since stroke might influence the accuracy of prediction models because spontaneous biological recovery efficiency is not considered [33–35]. The recovery potential of a certain treatment for patients might differ between those who are fitters of the “proportion recovery rule” and nonfitters [36]. Consequently, we believe that a well-defined recruitment criterion of participants in original modeling studies should be applied and reported to enhance the model interpretation [37].

### Predictors

To date, with the growing interest in predicting stroke rehabilitation outcome, variables such as age, initial motor impairment, stroke severity, biomarkers, and imaging data have been identified as significant factors for predicting stroke outcome [8, 38–40]. The candidate predictors selected in the included models varied. Demographic characteristics and clinical measures including age, sex, side of impairment, and baseline functional stages were commonly selected for analysis. Notably, treatment measures were not included as separate predictors during the variable selection process in most included studies; however, previous studies showed that a predictive model that does not include treatment as a predictor might omit intervention effects, thus leading to an inaccurate outcome [41, 42]. Although a concrete treatment strategy cannot be prospectively obtained before treatment, we recommend that a rehabilitation treatment plan tailored to patients could serve as a predictor in models to inform the potential recovery of individuals. In addition, given that the inconsistency among types of treatments for patients with stroke might increase the heterogeneity of results, we recommend that future studies report the details of structured interventions and facilitate the consistency of interventions.

### Outcome

Ideally, the outcome should be independently measured without information from predictors to reduce bias [17]. Due to the natural feature of the existing data source used in the included models, it is unclear whether the measurement of outcome was blindly recorded without information on predictors. Another concern is that nearly all the models included in this review assessed the outcome at post-treatment or discharge as a single endpoint, while other researchers propose that a single endpoint could not fully account for the improvement following rehabilitation if participants were recruited in wide time windows after stroke. The discharge timepoint is also inappropriate since it is often limited by local rehabilitation resources [34, 43]. Thus, we suggest that follow-up endpoints might be obtained to detect the longer-term benefits of a treatment and to ensure that the model’s predictive ability is as accurate as possible.

### Model analysis

The analysis process, which is also the main source of bias in the included models according to the PROBAST tool, could be improved in several aspects. First, a sufficient sample size for developing models, especially regression models, is usually based on the events per variable (EPV), which could be calculated by the number of candidate predictors [15, 44]. Generally, EPV less than 10 is considered insufficient, while the most adequate EPV is still being debated [45, 46]. The insufficient sample size may lead to overfitting in modeling studies [47–49]. Another aspect concerns how missing data were handled in the included models. Models that excluded patients with incomplete data rather than properly handled missing data might result in a selective sample and thus overestimated model performance [17, 50, 51]. Additionally, among the reviewed models, the most frequent method used during the predictor selection process was backward selection. However, overfitting should be quantified through internal validation if the model was developed based on an insufficient sample size [16, 17]. Previously published models that used univariate analysis to determine predictors should be avoided in future studies since this approach could lead to inaccurate predictor selection [16, 52, 53]. In future studies, researchers could combine both nonstatistical methods and statistical methods to identity the candidate predictors [16, 17].

Moreover, as for the method for developing models, the most frequently used method in the included studies was logistic regression, which is consistent with a recent review, indicating a preference for logistic regression models in this specific field [54]. Other machine learning algorithms, such as support vector machines, neural networks, and nearest neighbors, have only been used in studies published in recent years. Conventional regression models and novel machine learning models each have their own advantages. For example, while regression can enhance the interpretability of a model, its predictive performance may not be as good as that of novel machine learning algorithms, and vice versa [54]. Thus, future studies could explore other interpretability methods to explain the black-box model, such as one of the included studies in our review, which used four Explainable Artificial Intelligence (XAI) approaches to interpret the results of machine learning methods [26]. Finally, as for the model performance, in addition to the discrimination and calibration that should be appropriately assessed, a validation process is also essential to examine the reliability of models. Validation can be divided into internal and external validation. The former method, such as cross-validation and bootstrapping, attempts to quantify the model bias using the same database with model development. External validation aims to quantify any model bias through a database at the new participant level (e.g., from a different country, setting, recruitment time span), which is external to the model development database [15]. Although we mentioned four studies that had conducted external validation, three of them just randomly divided a single database into a development and a validation database, which is criticized as an inefficient external validation form. In this situation, the two split databases may differ by chance, and the sample size would be reduced [17, 29]. As it is increasingly recognized that the model predictive ability might vary across countries, participants, and periods, effective external validation is always recommended to present the possibility of heterogeneity in the predictive model [14, 20, 42, 43].

### Implications

With the development of machine learning in the field of medicine, there is a growing interest in the field of stroke rehabilitation. However, the number of high-quality models that meet the reporting rules and can be widely used is still limited, and future model development studies need to improve the quality of models in several ways and report the model development process according to the principle of transparency [55]. It is important to note that in the clinical setting, predictive models can only be used as a tool to assist physicians in decision-making, and the specific rehabilitation plan for the patient needs to be developed by the physician in the context of the patient’s actual condition.

### Limitations

This systematic review is limited by small sample sizes and suboptimal data sources for the included models, and thus the reported model performance may be overly optimistic. Moreover, due to large heterogeneity among studies, we did not conduct a meta-analysis, nor did we use quantitative methods to detect publication bias, so the results of this review should be treated with caution. Another limitation is that the rehabilitation treatment administered to patients varies across countries and rehabilitation settings, which may reduce the generalizability of the models.

## Conclusions

This review reveals potential gaps between ideal models and current models, and it is exciting to see that the included models have all shown relatively positive performances; however, existing modeling studies are constrained by small sample sizes and inconsistent results, indicating that there is still room for improvement. We believe that data sharing and coordinated efforts among countries could help future research in this area. Furthermore, as the number of proven significant predictors grows, prediction models should be dynamically updated. Applicable and reliable prediction models should help clinicians improve the implementation of patient-specific stroke rehabilitation treatment.

## Supporting information

S1 Checklist PRISMA 2020 checklist. (DOCX)

S1 Table Example of search strategy in PubMed. This is an example search strategy for PubMed.
(DOCX)

S2 Table Data extraction sheet. This is the details of the data extraction sheet.
(DOCX)
