# Supplementary material for: Machine learning in predicting outcomes for stroke patients following rehabilitation treatment: A systematic review
Source: PLoS One. 2023 Jun 28;18(6):e0287308. doi: 10.1371/journal.pone.0287308 (PMC10306189; doi:10.1371/journal.pone.0287308)
Supplement: S1 Table — This is an example search strategy for PubMed. (DOCX) [file pone.0287308.s002.docx]

**S1 Table. example of search strategy in Pubmed**

| #1 | "stroke"[MeSH Terms] |
| --- | --- |
| #2 | "brain infarction*"[Title/Abstract] OR "cerebrovascular disorder*"[Title/Abstract] OR  "cerebrovascular accident*"[Title/Abstract] OR "cerebral infarction*"[Title/Abstract] OR  "cerebrovascular disease*"[Title/Abstract] OR "cerebral haemorrhage"[Title/Abstract] OR  "cerebral hemorrhage"[Title/Abstract] OR "brain ischemia"[Title/Abstract] OR "brain  ischaemia"[Title/Abstract] OR "cerebral ischemia"[Title/Abstract] OR "cerebral  ischaemia"[Title/Abstract] |
| #3 | #1 OR #2 |
| #4 | "rehabilitation"[MeSH Terms] |
| #5 | "rehabilitative"[Title/Abstract] OR "neurorehabilitation"[Title/Abstract] OR "neurological  physiotherapy"[Title/Abstract] OR "neurological rehabilitation"[Title/Abstract] OR "stroke  rehabilitation"[Title/Abstract] OR "Physiotherapy"[Title/Abstract] OR "physical  rehabilitation"[Title/Abstract] OR "physical therapy"[Title/Abstract] OR "physical therapy  modalities"[Title/Abstract] OR "physiotherapy techniques"[Title/Abstract] OR "physiotherapy  rehabilitation"[Title/Abstract] OR "physical therapist"[Title/Abstract] OR  "physiotherapist"[Title/Abstract] OR "mobilization"[Title/Abstract] OR "activities of daily  living"[Title/Abstract] OR "functional activities"[Title/Abstract] OR "functional  training"[Title/Abstract] OR "exercise therapy"[Title/Abstract] OR "exercise"[Title/Abstract]  OR "ambulation"[Title/Abstract] OR "mobility"[Title/Abstract] OR "functional  assessment"[Title/Abstract] OR "occupational therapy"[Title/Abstract] OR "allied health  worker"[Title/Abstract] OR "allied health professional"[Title/Abstract] OR "occupational  therapist"[Title/Abstract] OR "virtual rehabilitation"[Title/Abstract] OR  "telerehabilitation"[Title/Abstract] OR "geriatric rehabilitation"[Title/Abstract] OR  "recreational therapy"[Title/Abstract] OR "vocational rehabilitation"[Title/Abstract] OR  "recovery of function"[Title/Abstract] |
| #6 | #4 OR #5 |
| #7 | "machine learning"[MeSH Terms] |
| #8 | "artificial intelligence"[Title/Abstract] OR "deep learning"[Title/Abstract] OR  "unsupervised machine learning"[Title/Abstract] OR "supervised machine  learning"[Title/Abstract] OR "neural network"[Title/Abstract] OR "support vector  machine"[Title/Abstract] OR "natural language processing"[Title/Abstract] OR "naive  bayes"[Title/Abstract] OR "bayesian learning"[Title/Abstract] OR "support  vector"[Title/Abstract] OR "random forest*"[Title/Abstract] OR "boosting"[Title/Abstract] OR  "algorithm"[Title/Abstract] OR "predictive analytics"[Title/Abstract] OR "physiologic  monitoring"[Title/Abstract] OR "prediction model"[Title/Abstract] OR "predictive  model"[Title/Abstract] |
| #9 | #7 OR #8 |
| #10 | #3 AND #6 AND #9 |
