# Supplementary material for: Machine learning in predicting outcomes for stroke patients following rehabilitation treatment: A systematic review
Source: PLoS One. 2023 Jun 28;18(6):e0287308. doi: 10.1371/journal.pone.0287308 (PMC10306189; doi:10.1371/journal.pone.0287308)
Supplement: S2 Table — This is the details of the data extraction sheet. (DOCX) [file pone.0287308.s003.docx]

**S2 Table. data extraction sheet**

| **Domain** | **Key items** |
| --- | --- |
| Study sets | Source of data |
|  | Population |
|  | Intended timing of model use |
|  | Setting |
|  | Number of centers |
| Participants | Inclusion criteria |
|  | Exclusion criteria |
|  | Details of treatment |
|  | Patient sex |
|  | Patient age |
| Outcome | Definition and method for measurement of outcome |
|  | Timing of outcome |
| Predictors | List of candidate predictors |
|  | Number of candidate predictors |
|  | Type of predictors |
|  | Definition and method for measurement of candidate predictors |
|  | Timing of predictors measurement |
|  | Handing of predictors in the model |
|  | Predictors in the final model |
| Sample size | Number of participants and number of outcomes/events |
|  | Number of outcomes/events in relation to the number of candidate predictors (Events Per Variable) |
| Model analysis | Modelling method |
|  | Method for selection of predictors for inclusion in multivariable modelling |
|  | Method for selection of predictors during multivariable modelling |
|  | Handling of missing data |
|  | Method(s) for validation (e.g. apparent, internal or external) and for optimism adjustment |
|  | Performance measures (calibration, discrimination, other) resulting from validation |
|  | Model presentation |
